# Tau Pathology Distribution in Alzheimer's disease Corresponds Differentially to Cognition-Relevant Functional Brain Networks

**DOI:** 10.3389/fnins.2017.00167

**Published:** 2017-03-31

**Authors:** Oskar Hansson, Michel J. Grothe, Tor Olof Strandberg, Tomas Ohlsson, Douglas Hägerström, Jonas Jögi, Ruben Smith, Michael Schöll

**Affiliations:** ^1^Clinical Memory Research Unit, Department of Clinical Sciences, Lund UniversityMalmö, Sweden; ^2^Memory Clinic, Skåne University HospitalMalmö, Sweden; ^3^German Center for Neurodegenerative DiseasesRostock, Germany; ^4^Department of Radiation Physics, Skåne University HospitalLund, Sweden; ^5^Department of Clinical Neurophysiology, Skåne University HospitalLund, Sweden; ^6^Department of Clinical Physiology and Nuclear Medicine, Skåne University HospitalLund, Sweden; ^7^Department of Neurology, Skåne University HospitalLund, Sweden; ^8^MedTech West and the Department of Psychiatry and Neurochemistry, University of GothenburgGothenburg, Sweden

**Keywords:** Alzheimer's disease, functional brain networks, tau, positron emission tomography

## Abstract

Neuropathological studies have shown that the typical neurofibrillary pathology of hyperphosphorylated tau protein in Alzheimer's disease (AD) preferentially affects specific brain regions whereas others remain relatively spared. It has been suggested that the distinct regional distribution profile of tau pathology in AD may be a consequence of the intrinsic network structure of the human brain. The spatially distributed brain regions that are most affected by the spread of tau pathology may hence reflect an interconnected neuronal system. Here, we characterized the brain-wide regional distribution profile of tau pathology in AD using ^18^F-AV 1451 tau-sensitive positron emission tomography (PET) imaging, and studied this pattern in relation to the functional network organization of the human brain. Specifically, we quantified the spatial correspondence of the regional distribution pattern of PET-evidenced tau pathology in AD with functional brain networks characterized by large-scale resting state functional magnetic resonance imaging (rs-fMRI) data in healthy subjects. Regional distribution patterns of increased PET-evidenced tau pathology in AD compared to controls were characterized in two independent samples of prodromal and manifest AD cases (the Swedish BioFINDER study, *n* = 44; the ADNI study, *n* = 35). In the BioFINDER study we found that the typical AD tau pattern involved predominantly inferior, medial, and lateral temporal cortical areas, as well as the precuneus/posterior cingulate, and lateral parts of the parietal and occipital cortex. This pattern overlapped primarily with the dorsal attention, and to some extent with higher visual, limbic and parts of the default-mode network. PET-evidenced tau pathology in the ADNI replication sample, which represented a more prodromal group of AD cases, was less pronounced but showed a highly similar spatial distribution profile, suggesting an earlier-stage snapshot of a consistently progressing regional pattern. In conclusion, the present study indicates that the regional deposition of tau aggregates in AD predominantly affects higher-order cognitive over primary sensory-motor networks, but does not appear to be specific for the default-mode or related limbic networks.

## Introduction

The brain can be subdivided into a number of intrinsic connectivity networks (ICNs), which have been established mainly through interpretation of large resting-state functional magnetic resonance imaging (rs-fMRI) datasets (Power et al., 2011; Yeo et al., 2011). These networks have been found to be disrupted in a distinct manner in Alzheimer's disease (AD), directly affecting cognitive performance (Pievani et al., 2011). The accumulation of fibrillar amyloid-β (Aβ), one of the major traits of AD pathogenesis, has consistently been demonstrated to follow spatial patterns (Braak and Braak, 1991; Villeneuve et al., 2015) that overlap strongly with the so-called default-mode network (DMN; Buckner et al., 2009; Sperling et al., 2009), a network of brain regions generally activated in “task-negative” states but also during autobiographical and introspective tasks (Greicius et al., 2003, 2004). However, a recent study generally confirming these findings, also observed considerable spatial overlap of Aβ accumulating regions with other ICNs, such as a frontoparietal-control network (FPN) and the dorsal-attention network (DAN; Grothe and Teipel, 2016). This study further found substantial dissociation between correspondence of imaging-derived measures of Aβ pathology, measures of neurodegeneration proxied by glucose hypometabolism and gray matter atrophy, and ICNs (Grothe and Teipel, 2016).

Hyperphosphorylated tau, another misfolded protein to accumulate in AD, is known to be closer related to neurodegeneration and cognitive impairment than Aβ (Serrano-Pozo et al., 2011; Nelson et al., 2012). The recent introduction of tau-sensitive positron emission tomography (PET) ligands binding to paired-helical filaments of tau protein has enabled the *in vivo* assessment of hyperphosphorylated tau pathology (Xia et al., 2013; Marquié et al., 2015). Neuropathological studies have established a rather consistent spatial pattern of progressing tau pathology that has been categorized into so-called Braak stages (Braak and Braak, 1991; Braak et al., 2006). These nested stages of progressing tau pathology could recently be replicated *in vivo* using tau PET (Schwarz et al., 2016; Schöll et al., 2016).

Studies in cell and transgenic mouse models further suggest that tau in its harmful hyperphosphorylated form likely spreads in a trans-synaptic manner along neuronal networks, eventually causing synaptic damage, functional disruption, and neurodegeneration of the affected networks (Fox et al., 2011; Liu et al., 2012; Spires-Jones and Hyman, 2014; Menkes-Caspi et al., 2015).

In the present study, we aimed at exploring the spatial overlap of pre-defined functional brain networks and the extent of neurofibrillary tau pathology assessed with ^18^F-AV-1451 PET in prodromal and dementia stages of AD, also answering the question whether tau pathology preferrably affects the DMN over other ICNs.

## Methods

### Participants

We studied samples from two independent cohorts. The first sample was recruited from the prospective and longitudinal Swedish BioFINDER study (further information available at: www.BioFINDER.se). We included data from 17 cognitively healthy elderly control (HC) participants and 27 AD patients with prodromal (MCI due to AD, *n* = 11) or clinically manifest disease (AD dementia, *n* = 16). Inclusion criteria for HC were: (1) aged ≥ 60 years old, (2) scored 28–30 points on the Mini-Mental State Examination (MMSE) at the screening visit, (3) absence of cognitive symptoms as evaluated by a physician, (4) fluent in Swedish, (5) did not fulfill the criteria of MCI or any dementia; with the following exclusion criteria: (1) significant systemic illness making it difficult to participate, (2) presence of significant neurologic or psychiatric disease (*e.g*., stroke, Parkinson's disease, multiple sclerosis, major depression), (3) significant substance abuse. Inclusion criteria for MCI due to AD were: (1) age 40–100 years, (2) referred to the memory clinics due to cognitive symptoms experienced by the patient and/or informant, (3) MMSE score of 24–30, (4) objective impairment according to neuropsychological testing, (5) no fulfillment of the criteria for any dementia disorder (major neurocognitive disorder) according to DSM-V, (6) abnormal cerebrospinal fluid (CSF) Aβ42 indicative of prodromal AD and (7) fluency in Swedish; and the exclusion criteria were: (1) cognitive impairment that without doubt could be explained by another condition (other than prodromal AD) and (2) severe somatic disease. AD dementia patients met the DSM-IIIR criteria for dementia (American Psychiatric Association and American Psychiatric Association Work Group to Revise DSM-III., 1987) as well as the NINCDS-ADRDA criteria for AD (McKhann et al., 1984). Exclusion criteria were significant systemic illness and significant alcohol abuse. AD diagnoses were confirmed by physicians who were blinded to any PET and CSF data. We only recruited AD patients with a late-onset amnestic clinical presentation to ensure a typical pattern of tau ligand uptake as differences in ligand retention between different AD variants had previously been reported (Ossenkoppele et al., 2016). All participants provided written informed consent to participate in the study according to the Declaration of Helsinki, ethical approval was given by the Ethics Committee of Lund University, Lund, Sweden, and all methods were carried out in accordance with the approved guidelines. Approval for PET imaging was obtained from the Swedish Medicines and Products Agency and the local Radiation Safety Committee at Skåne University Hospital, Sweden.

The second sample was recruited from the ADNI2 cohort, an extension of the original ADNI1 and ADNI-GO studies. ADNI (Alzheimer's Disease Neuroimaging Initiative) is a multisite longitudinal biomarker study that has enrolled over 1,500 cognitively normal older individuals, people with early or late amnestic MCI, and people with early AD (www.adni-info.org). Detailed inclusion and exclusion criteria for the diagnostic categories can be found on the ADNI website (http://adni.loni.usc.edu/methods/). Briefly, healthy subjects had MMSE scores between 24 and 30 (inclusive), a clinical dementia rating (CDR) = 0, were non-depressed, non-MCI, and non-demented. MCI subjects had MMSE scores between 24 and 30 (inclusive), a subjective memory concern reported by subject, informant, or clinician, objective memory loss measured by education adjusted scores on delayed recall, a CDR = 0.5, absence of significant levels of impairment in other cognitive domains, essentially preserved activities of daily living, and an absence of dementia. Subjects with AD dementia had initial MMSE scores between 20 and 26 (inclusive), a CDR = 0.5 or 1.0, and fulfill NINCDS-ADRDA criteria for clinically probable Alzheimer's disease (McKhann et al., 1984). For the present study, we recruited data from 19 HC, 26 prodromal, and four patients with manifest AD.

Diagnostic groups were dichotomized into β-amyloid-positive (Aβ+) and -negative (Aβ−) subgroups, based on ^18^F-flutemetamol (BioFINDER) or ^18^F-AV45 (ADNI) PET evidence of global Aβ pathology indicative of AD. For ADNI subjects, cortex-to-whole cerebellum AV45 standard uptake value ratios (SUVR) had been calculated and made available on the ADNI server by the Jagust Lab, UC Berkeley. Aβ-positivity was established using the recommended threshold for cross-sectional analyses of SUVR ≥ 1.11 (Landau et al., 2013). For BioFINDER subjects, global ^18^F-flutemetamol SUVR had been calculated using a composite whole cerebellum, the pons/brainstem region, and eroded cortical white matter reference region; a cutoff of SUVR ≥ 0.79 was derived using a mixture modeling analysis in a large BioFINDER cohort (*n* = 406) to describe Aβ-positivity.

Given that we aimed at examining a typical late-onset amnestic AD type, and with all recruited BioFINDER MCI and AD subjects being Aβ-positive, Aβ-negative subjects were omitted from the ADNI2 sample, which resulted in a final ADNI sample size of 19 HC and 16 prodromal dementia and clinically manifest AD patients (12 Aβ + MCI, 4 Aβ + AD; Albert et al., 2011; McKhann et al., 2011; Sperling et al., 2011).

### Image acquisition

All BioFINDER subjects underwent structural magnetic resonance imaging (MRI) on a Siemens Tim Trio 3T scanner (Siemens Medical Solutions, Erlangen, Germany). High resolution T1-weighted anatomical magnetization-prepared rapid gradient echo (MPRAGE) images (TR = 1950 ms TE = 3.4 ms, 1 mm isotropic voxels and 176 slices) were acquired for PET image co-registration, processing, and template normalization.

^18^F-AV-1451 PET scans were performed on a GE Discovery 690 PET scanner (General Electric Medical Systems) as dynamic scans using LIST-mode 80–120 min after a bolus injection of 370 MBq of ^18^F-AV-1451. Low-dose CT-scans for attenuation correction were performed immediately prior to the PET scans. PET data were reconstructed into 5 min frames using an iterative Vue Point HD algorithm with six subsets, 18 iterations with 3 mm filter, and no time-of-flight correction. The dynamic scans were motion corrected using AFNI's 3dvolreg (Cox, 1996), time-averaged, and rigidly co-registered to the skull-stripped MRI scan.

For ADNI participants, T1-weighted MPRAGE MR images were acquired on multiple 3T scanners. ^18^F-AV-1451 PET scans were also acquired on several different scanners at multiple sites. Subjects were examined for 30 min (6 × 5 min frames) starting at 75 min post-injection of a 370 MBq/kg bolus. Standardized image pre-processing is applied to all original ADNI scans (see http://adni.loni.usc.edu/methods for details).

### Image processing

Preprocessed ADNI image data was downloaded from the ADNI image database (https://ida.loni.usc.edu) and further processed in the same manner as BioFINDER image data (see below).

All image data was thus processed at Lund University employing an in-house developed pipeline. The MR scans were normalized to a common MNI152 space (Montreal Neurological Institute) with a diffeomorphic transform using the Advanced Normalization Tools (ANTs) toolbox (Avants et al., 2014) for further use in the PET processing pipeline. Cortical reconstruction and volumetric segmentation and parcellation were performed with Freesurfer v5.3 (http://surfer.nmr.mgh.harvard.edu). Reconstructed data sets were visually inspected for inaccuracies, and major segmentation errors were manually corrected. The Freesurfer parcellation in the MR space of the anatomical scan was then applied to the processed, coregistered, and time-averaged PET image to extract reference regional uptake values.

We created BioFINDER ^18^F-AV-1451 standardized uptake value (SUV) PET images based on mean uptake over 80–120 min postinjection normalized to uptake in a gray matter masked cerebellum reference region to create voxelwise SUV ratio (SUVR) images in each participant's MRI native space. ADNI ^18^F-AV-1451 SUVR images were created based on mean uptake over 80–100 min post-injection and intensity normalized using the same cerebellar reference region as for the BioFINDER data. All PET images were then spatially normalized to MNI152 space, employing the ANTs spatial transformation parameters derived from the co-registered MR scans, and smoothed with a 8 mm FWHM Gaussian filter. Using FSL (v5.0.6, http://fsl.fmrib.ox.ac.uk), we finally created a mean and a standard deviation image based on the respective control group's ^18^F-AV-1451 images in MNI152 standard space and calculated individual Z-score maps for each patient (Z-score = (individual value—control mean)/control standard deviation; Chételat et al., 2008; Grothe and Teipel, 2016).

### Data analysis

Analysis of the network-specificity of the AD-typical *in vivo* distribution of tau deposits as imaged by ^18^F-AV-1451 PET closely followed the approach of a previously published study assessing the network-specificity of AD-related amyloid deposition, hypometabolism, and gray matter atrophy (Grothe and Teipel, 2016). Briefly, this approach quantitatively assesses the correspondence of the cortex-wide pathologic imaging pattern with functional networks in the human brain as defined by standardized maps of ICN derived from large-scale resting-state rs-fMRI data of a healthy adult population (Yeo et al., 2011). Quantitative metrics include the mean Z-score within each ICN template, reflecting the extent of tau deposition in relation to control values, as well as a goodness-of-fit (GOF) score for each ICN template quantifying the spatial correspondence of the cortex-wide tau deposition pattern with the functional network topography. GOF scores are calculated as the difference between the mean Z-score value of voxels falling within a given ICN template and the mean Z-score value of cortical voxels outside the ICN template (Greicius et al., 2004; Lehmann et al., 2013). Thus, a positive GOF-score indicates a relative preference of cortex-wide tau deposition to occur within the respective ICN, whereas a uniform distribution of tau deposition across the cortex would result in GOF-scores close to zero for all ICNs.

One sample *t*-tests were used to assess the significance of ICN-specific increases in tau deposition in the patient groups compared to the respective control groups (mean Z-scores per ICN), as well as the significance of the spatial correspondence of the AD-typical tau deposition with a given ICN (positive GOF-scores). Statistical significance was set at *p* < 0.05 (two-tailed), Bonferroni-corrected for the number of networks assessed.

**Figure 1:**
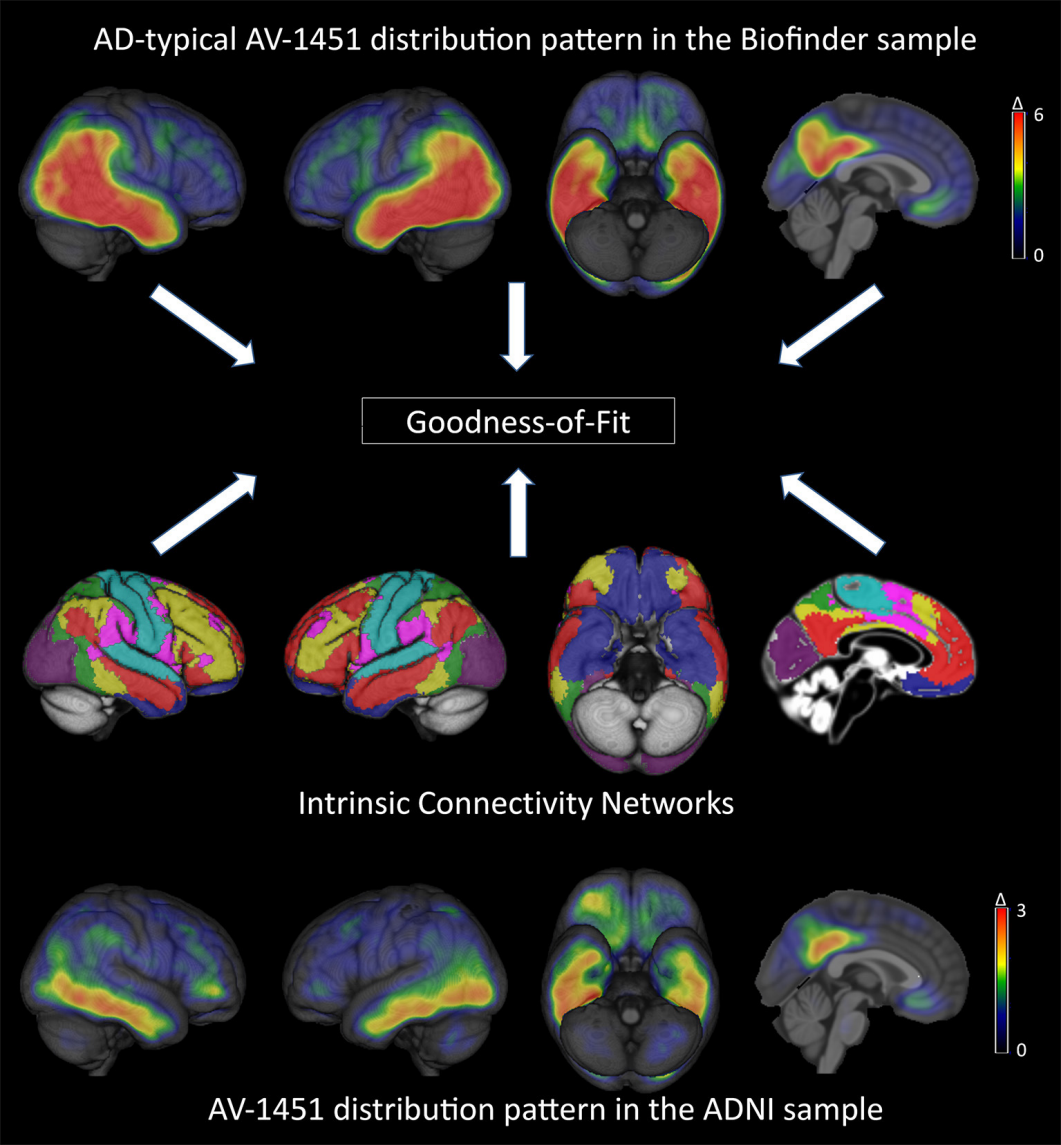
**Brain-wide patterns of increased tau deposition in the BioFINDER and ADNI cohorts and illustration of the standardized intrinsic connectivity networks (for details see Supplementary Figure **1**)**. The top and bottom row images represent mean Z-score images of the BioFINDER and ADNI patient samples. Note the different scales used for color-coded visualization of mean Z-score values in the BioFINDER and ADNI patient samples.

In our primary analysis we used an ICN definition based on a recently published functional parcellation scheme of the human brain into seven major ICNs, including the 1, DMN; 2, frontoparietal-control network (FPN); 3, dorsal attention network (DAN); 4, ventral attention network (VAN); 5, limbic network (LIM); 6, visual network (VIS) and 7, somatomotor network (SMN; Figure 1, Supplementary Figure 1; Yeo et al., 2011; https://sites.google.com/site/yeoyeo02/software). While these principal large-scale ICNs are consistently reproduced in rs-fMRI based functional parcellations across several independent cohorts and using diverse parcellation methods (Greicius et al., 2003; Damoiseaux et al., 2006; Fox et al., 2006; Cohen et al., 2008; Kahn et al., 2008; Vincent et al., 2008; Smith et al., 2009; Bellec et al., 2010; Power et al., 2011; Jones et al., 2012; Das et al., 2015; Pascual et al., 2015), there is currently no established way of unambiguously defining the most appropriate number of separate connectivity modules within the brain's functional connectivity architecture, and at higher parcellation resolutions, the principal large-scale ICNs typically fractionate into further submodules (Andrews-Hanna et al., 2010; Power et al., 2011). Thus, in a secondary analysis we assessed the network-specificity of the AD-typical tau deposition pattern using a more fine-grained parcellation scheme into 17 functional subnetworks (Yeo et al., 2011): 1, Higher visual; 2, Primary visual; 3, Dorsal SMN; 4, Ventral SMN-auditory; 5, Posterior DAN; 6, Frontal eye field-DAN; 7, Posterior VAN; 8, Anterior VAN; 9, Temporal pole-Anterior medial temporal lobe (MTL) LIM; 10, Orbitofrontal LIM; 11, Precuneus non-DMN; 12, FPN component 1; 13, FPN component 2; 14, Lateral-temporal DMN/language; 5, Posterior MTL-retrosplenial DMN; 16, Midline DMN; 17, Anterior DMN (these codes are consequently used in graphs and tables).

The spatial correspondence between regional tau distribution profiles in the independent BioFINDER and ADNI samples was assessed using Pearson's correlation across all cortical voxels of the mean Z-score maps from the respective cohorts (Buckner et al., 2009).

## Results

### Participants

Demographics for all participants are displayed in Table 1. There was no statistically significant difference in age between the BioFINDER groups, but the ADNI HC were significantly younger than the ADNI patient group. Patient groups in both BioFINDER and ADNI performed significantly worse on the MMSE when compared to the respective HC.

**Table 1:** **Demographics**.

	**Age (y)**	**Sex (M/F)**	**Education (y)**	**MMSE**	**% prodromal AD patients**
HC BioFINDER (*n* = 17)	73.1 ± 6.1	9/8	12.2 ± 4.5	29.4 ± 0.9	
Patients BioFINDER (*n* = 27)	73.8 ± 5.7	18/9	12.0 ± 3.9	21.9 ± 5.5^*^	41
HC ADNI (*n* = 19)	73.7 ± 6.3	7/12	15.7 ± 2.6	28.6 ± 1.6	
Patients ADNI (*n* = 16)	81.0 ± 6.1^#^	12/4	16.1 ± 3.2	25.8 ± 2.5^#^	75

*Demographics of all participants, all values are mean ±standard deviation unless stated otherwise. HC, Healthy controls; MMSE, Mini mental state examination*.

* significantly different from HC (BioFINDER);

# *Significantly different from HC (ADNI); p < 0.05; Kruskal-Wallis H test with Dunn's post-hoc test*.

### Consistent brain-wide pattern of increased ^18^F-AV-1451 uptake in AD

**Figure 2:**
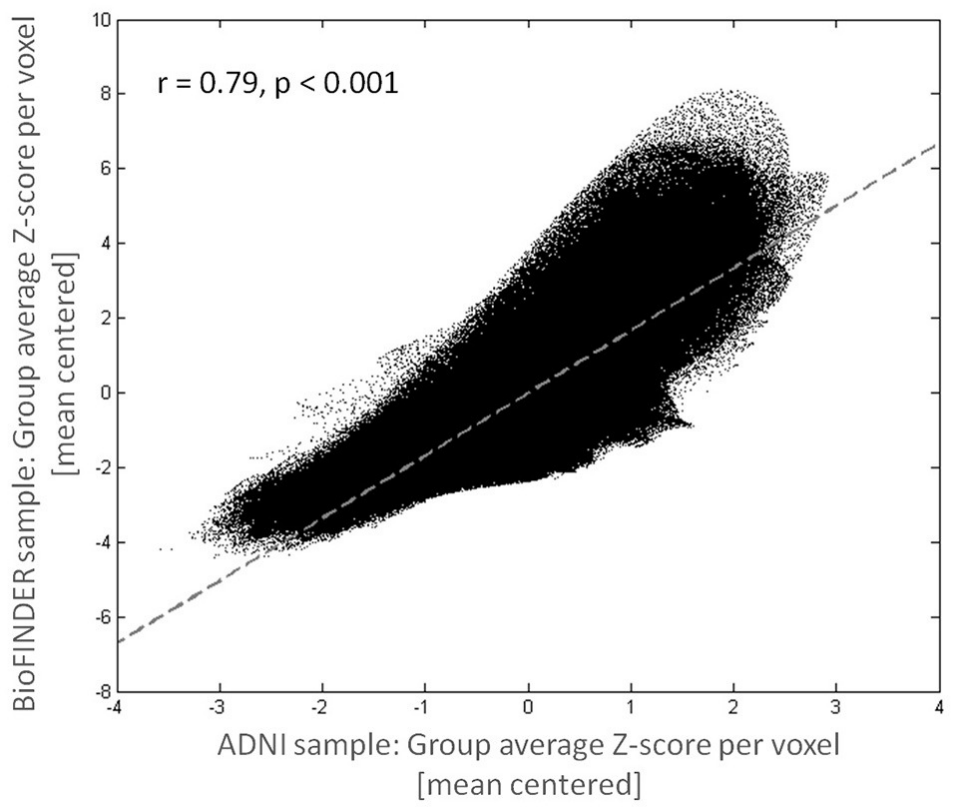
**Consistency of tau deposition pattern across cohorts**. Mean voxelwise cortical Z-scores for the BioFINDER and ADNI sample were highly correlated.

Figure 1 shows mean Z-score images of the AD patients from the BioFINDER and the ADNI cohort, respectively. The BioFINDER sample with its higher proportion of manifest AD dementia cases displayed clear-cut ^18^F-AV-1451 uptake in the lateral and inferior temporal lobes with partial involvement of the lateral parietal and occipital cortices, as well as in the precuneus and posterior cingulate. The ADNI sample with its greater proportion of prodromal AD cases exhibited less pronounced ligand uptake, albeit demonstrating a very similar spatial distribution profile across all cortical voxels (*r* = 0.79, *p* < 0.001; Figures 1, 2).

### The brain-wide pattern of increased ^18^F-AV-1451 uptake in AD overlaps with several functional brain networks related to higher cognition

#### Biofinder sample

**Figure 3:**
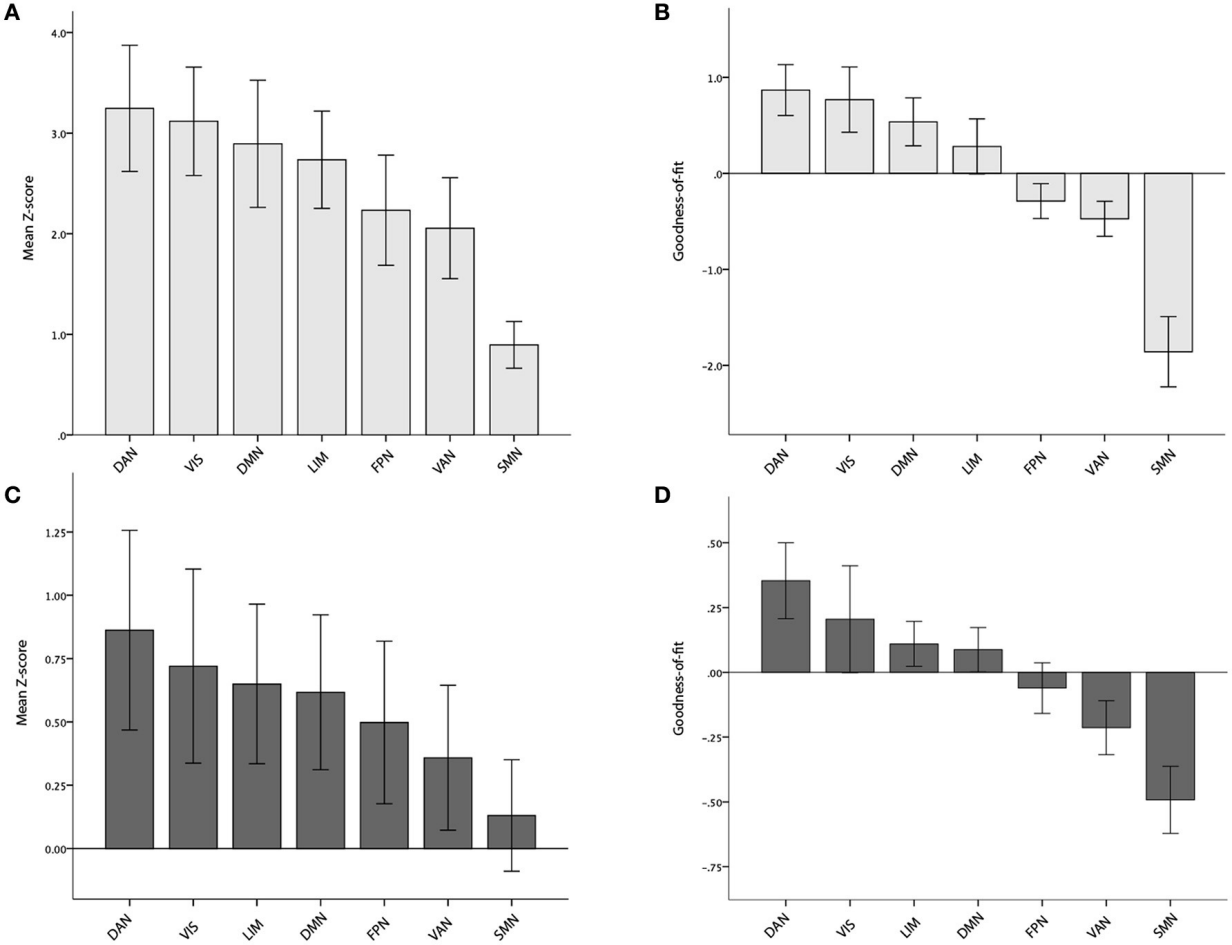
**Mean Z- and Goodness-of-fit scores for each of the seven main networks in the BioFINDER (A,B)** and ADNI **(C,D)** samples. Error bars represent 1 standard error.

Increased AV-1451 uptake was observed in all seven main ICNs at a corrected alpha level = 0.05/7 = 0.007 (Z-scores different from 0, Table 2, Figure 3A), but AV-1451 retention was not homogeneously distributed across these networks. The gradient of severity of tau accumulation followed the order: DAN > VIS > DMN > LIM > FPN > VAN > SMN. Accordingly, positive GOF-scores, indicating a preferential tau accumulation in relation to brain-wide uptake, were observed for the DAN (*p* < 0.001), with trends (*p* < 0.05) for the VIS (*p* = 0.03), and the DMN (*p* = 0.04; Table 2, Figure 3B).

**Table 2:** **Z- and Goodness-of-fit scores for AV-1451 uptake in seven large-scale networks in the BioFINDER sample**.

			**One-sample *t*-test, test value = 0**	
	**Mean**	**SD**	***t***	**Sig. (2-tailed)**
z_DAN	3.246	3.2579	5.177	0.000
z_VIS	3.118	2.8011	5.784	0.000
z_DMN	2.894	3.2823	4.581	0.000
z_LIM	2.736	2.5115	5.660	0.000
z_FPN	2.233	2.8461	4.077	0.000
z_VAN	2.055	2.6086	4.093	0.000
z_SMN	0.895	1.2064	3.856	0.001
GOF_DAN	0.868	1.3779	3.273	0.003
GOF_VIS	0.769	1.7645	2.264	0.032
GOF_DMN	0.537	1.2987	2.149	0.041
GOF_LIM	0.281	1.4948	0.976	0.338
GOF_FPN	−0.288	0.9409	−1.592	0.123
GOF_VAN	−0.474	0.9514	−2.587	0.016
GOF_SMN	−1.859	1.8978	−5.089	0.000

**Figure 4:**
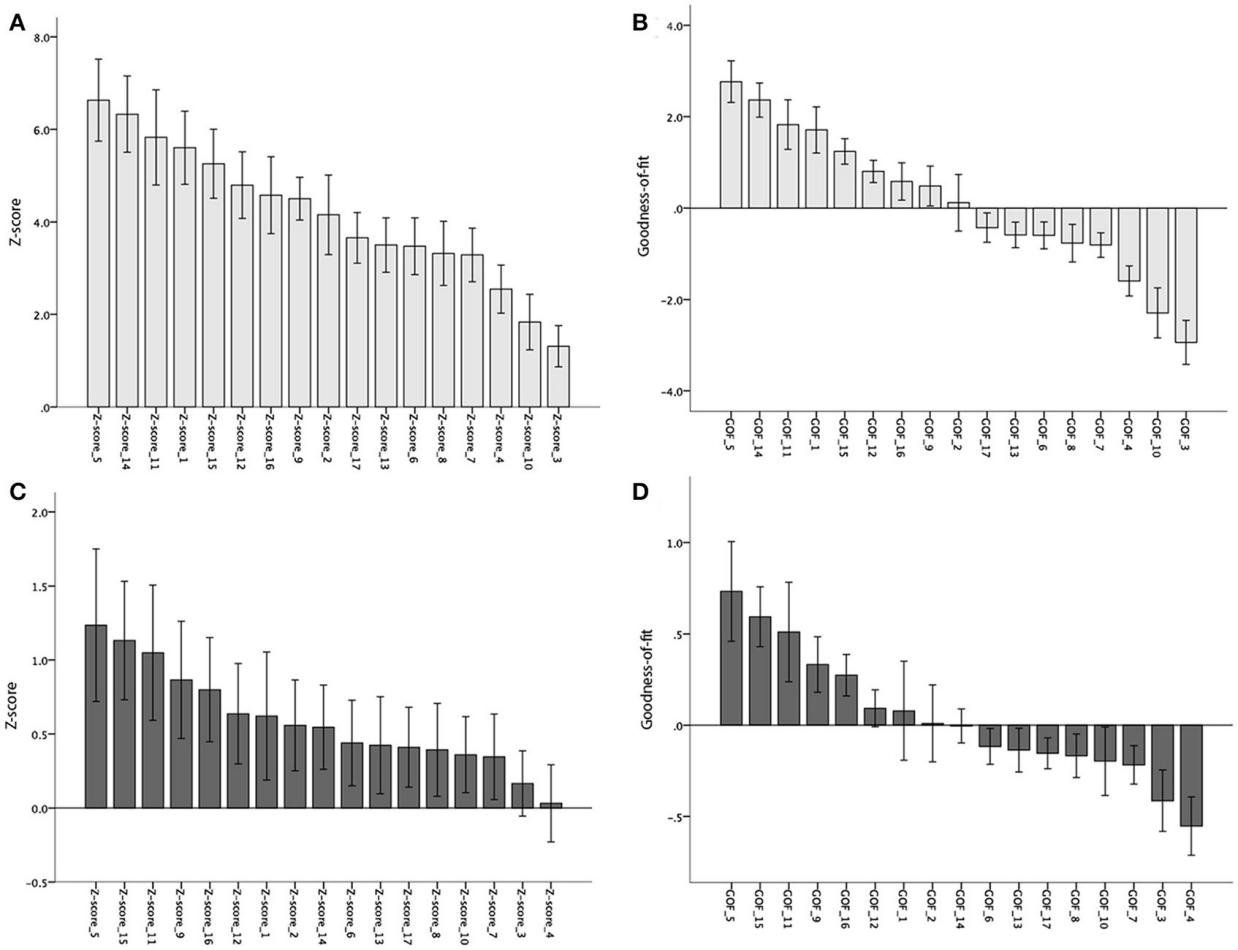
**Mean Z- and Goodness-of-fit scores for each of the 17 sub-networks in the BioFINDER (A,B)** and ADNI **(C,D)** samples. Error bars represent 1 standard error.

Fifteen of the 17 subnetworks showed significantly increased AV-1451 uptake at a corrected alpha level = 0.05/17 = 0.003 (Z-scores different from 0, Table 3, Figure 4A). Most clear AV-1451 uptake was observed in nodes of the posterior DAN (5), a lateral temporal/language subnetwork of the DMN (14), a medial parietal/precuneal component of the FPN (11), a higher visual network (1), and a posterior MTL/retrosplenial subnetwork of the DMN (15) (all z-score >5). Least pronounced uptake was seen in the dorsal and ventral SMN (3, 4) and the orbitofrontal node of the limbic network (10) (all z-scores ≤2.5).

**Table 3:** **Z- and Goodness-of-fit scores for AV-1451 uptake in 17 sub-networks in the BioFINDER sample**.

			**One sample *t*-test, test value = 0**	
	**Mean**	**SD**	***t***	**Sig. (2-tailed)**
Z-score_5	6.631	4.6036	7.485	0.000
Z-score_14	6.330	4.2898	7.667	0.000
Z-score_11	5.829	5.3535	5.658	0.000
Z-score_1	5.605	4.1096	7.087	0.000
Z-score_15	5.258	3.8827	7.036	0.000
Z-score_12	4.794	3.7445	6.652	0.000
Z-score_16	4.576	4.3093	5.518	0.000
Z-score_9	4.504	2.4032	9.738	0.000
Z-score_2	4.155	4.4634	4.838	0.000
Z-score_17	3.657	2.8548	6.655	0.000
Z-score_13	3.501	3.0659	5.934	0.000
Z-score_6	3.476	3.1934	5.656	0.000
Z-score_8	3.321	3.6034	4.789	0.000
Z-score_7	3.287	3.0157	5.664	0.000
Z-score_4	2.546	2.6970	4.905	0.000
Z-score_10	1.835	3.1176	3.058	0.005
Z-score_3	1.313	2.3167	2.944	0.007
GOF_5	2.767	2.3600	6.092	0.000
GOF_14	2.363	1.9453	6.313	0.000
GOF_11	1.825	2.8125	3.372	0.002
GOF_1	1.709	2.6350	3.371	0.002
GOF_15	1.239	1.4417	4.467	0.000
GOF_12	0.804	1.2682	3.293	0.003
GOF_16	0.583	2.1319	1.421	0.167
GOF_9	0.484	2.2731	1.106	0.279
GOF_2	0.118	3.2155	0.191	0.850
GOF_17	−0.428	1.6645	−1.336	0.193
GOF_13	−0.587	1.4408	−2.117	0.044
GOF_6	−0.599	1.5241	−2.041	0.052
GOF_8	−0.769	2.1268	−1.879	0.072
GOF_7	−0.808	1.3893	−3.024	0.006
GOF_4	−1.594	1.6980	−4.879	0.000
GOF_10	−2.295	2.8371	−4.202	0.000
GOF_3	−2.938	2.5030	−6.100	0.000

Statistically significant positive GOF scores (*p* < 0.003) were observed for a total of six subnetworks: posterior DAN (5), lateral-temporal language (14), precuneus non-DMN (11), higher visual (1), posterior MTL-retrosplenial (15), and medial parietal and lateral frontoparietal components (12; Table 3, Figure 4B).

#### ADNI sample

At a corrected alpha-level of *p* < 0.007, we observed no significantly increased AV-1451 uptake compared to the control group within any of the seven large-scale brain networks. However, in accordance with the very high similarity in the voxel-wise regional distribution profile (Figure 2), the overall rank order of network involvement was very similar to the pattern in the BioFINDER sample (DAN > VIS > LIM > DMN > FPN > VAN > SMN), and trends for increased AV-1451 uptake compared to the control group were noted in the DAN (*p* = 0.045), LIM (*p* = 0.057), and DMN (*p* = 0.062; Table 4, Figure 3C). The only network that showed a trend for a significantly positive GOF score was the DAN (*p* = 0.03; Table 4, Figure 3D).

**Table 4:** **Z- and Goodness-of-fit scores for AV-1451 uptake in seven large-scale networks in the ADNI sample**.

			**One sample *t*-test, test value = 0**	
	**Mean**	**SD**	***t***	**Sig. (2-tailed)**
z_DAN	0.862	1.578	2.186	0.045
z_VIS	0.720	1.533	1.879	0.080
z_LIMB	0.650	1.260	2.063	0.057
z_DMN	0.617	1.223	2.019	0.062
z_FPN	0.498	1.284	1.551	0.142
z_VAN	0.358	1.144	1.252	0.230
z_SMN	0.130	0.881	0.592	0.563
GOF_DAN	0.354	0.585	2.418	0.029
GOF_VIS	0.205	0.825	0.995	0.335
GOF_LIMB	0.110	0.346	1.269	0.224
GOF_DMN	0.087	0.343	1.019	0.324
GOF_FPN	−.061	0.391	−0.619	0.545
GOF_VAN	−0.213	0.416	−2.055	0.058
GOF_SMN	−0.492	0.518	−3.796	0.002

At a corrected alpha-level of *p* < 0.003, none of the 17 subnetworks showed significantly increased tau uptake compared to the control group. However, similar to the findings in the BioFINDER sample, trends (*p* < 0.05) were observed for the posterior DAN (5), a medial parietal/precuneal component of the FPN (11), as well as the temporal pole-anterior MTL limbic subnetwork (9), and posterior MTL/retrosplenial (15) and midline (16) subnetworks of the DMN. Further, similar to the findings in the BioFINDER sample, the weakest uptake was seen in the dorsal and ventral somatomotor networks (3, 4), the posterior VAN (7), and the orbitofrontal node of the limbic network (10) (Table 5, Figure 4C). A significant positive GOF score was only observed for the posterior DAN (*p* = 0.003; Table 5, Figure 4D).

**Table 5:** **Z- and Goodness-of-fit scores for AV-1451 uptake in 17 sub-networks in the ADNI sample**.

			**One sample t-test, test Value = 0**	
	**Mean**	**SD**	***t***	**Sig. (2-tailed)**
Z-score_5	1.235	2.059	2.398	0.030
Z-score_15	1.131	1.601	2.825	0.013
Z-score_11	1.049	1.828	2.294	0.037
Z-score_9	0.866	1.582	2.189	0.045
Z-score_16	0.800	1.408	2.272	0.038
Z-score_12	0.637	1.358	1.875	0.080
Z-score_1	0.622	1.730	1.438	0.171
Z-score_2	0.559	1.227	1.821	0.089
Z-score_14	0.546	1.141	1.914	0.075
Z-score_6	0.440	1.155	1.522	0.149
Z-score_13	0.424	1.312	1.293	0.216
Z-score_17	0.411	1.078	1.523	0.148
Z-score_8	0.393	1.257	1.251	0.230
Z-score_10	0.360	1.029	1.400	0.182
Z-score_7	0.346	1.155	1.199	0.249
Z-score_3	0.166	0.882	0.752	0.464
Z-score_4	0.032	1.041	0.122	0.905
GOF_5	0.732	1.092	2.683	0.017
GOF_15	0.594	0.656	3.622	0.003
GOF_11	0.510	1.090	1.873	0.081
GOF_9	0.332	0.606	2.195	0.044
GOF_16	0.274	0.455	2.407	0.029
GOF_12	0.093	0.406	0.917	0.373
GOF_1	0.079	1.084	0.291	0.775
GOF_2	0.010	0.844	0.046	0.964
GOF_14	−0.004	0.373	−0.041	0.968
GOF_6	−0.116	0.394	−1.179	0.257
GOF_13	−0.136	0.480	−1.134	0.275
GOF_17	−0.154	0.337	−1.827	0.088
GOF_8	−0.167	0.477	−1.398	0.183
GOF_10	−0.197	0.749	−1.050	0.310
GOF_7	−0.217	0.420	−2.071	0.056
GOF_3	−0.413	0.672	−2.458	0.027
GOF_4	−0.551	0.640	−3.445	0.004

## Discussion

In the present study, we examined the spatial distribution of ^18^F-AV-1451 retention, likely representing the presence of hyperphosporylated tau pathology, in the brains of prodromal and clinically manifest AD patients in relation to the spatial extent of predefined templates of functional brain networks. In the past years, emerging evidence for a consistent regional deposition pattern of *in vivo* tau PET ligands in AD supports a typical involvement of the inferior and lateral temporal lobes, precuneus and posterior cingulate, as well as occipital and lateral parietal lobes (Brier et al., 2016; Ishiki et al., 2015; Johnson et al., 2016; Ossenkoppele et al., 2016; Schwarz et al., 2016). Our voxel-wise (average Z-score) maps of regional AV-1451 distribution reflect these reports of regional tau PET-ligand retention in independent AD cohorts. Accordingly, the regional tau distribution profiles were very similar between the independent BioFINDER and ADNI cohorts of our study, despite obvious differences in recruitment criteria, PET scanning platforms, disease severity, and age. The ADNI sample featuring a greater proportion of prodromal AD cases consequently demonstrated a slightly alleviated AV-1451 signal when compared to the BioFINDER sample.

Previous research has suggested that AD pathology and related brain atrophy predominantly target the DMN (Seeley et al., 2009), a finding we could not confirm for the *in vivo* measures of AD-related tau pathology in our study. This is in line with a previous investigation of network-specific atrophy measures in AD (Grothe and Teipel, 2016), indicating little specificity for the entire DMN, but rather a distinct spatial overlap with an anterior limbic subnetwork. Here, we found increased tau deposition in limbic and DMN network components, but especially posterior cortical networks (dorsal attention and higher visual networks). A recent publication reported a significant correlation between regional AV-1451 ligand uptake and FDG-PET measures of neurodegeneration across AD patients of differing clinical phenotypes (Ossenkoppele et al., 2015, 2016). However, spatial correspondence of the two imaging modalities was far from being complete (*r* = −0.49 to −0.60), and future studies will have to further explore similarities and differences in the *in vivo* spatial distribution profiles of AD-related tau pathology and neurodegeneration, as well as their respective relations to the spatial topography of intrinsic brain networks (Sepulcre et al., 2016).

The posterior cortical predominance of *in vivo* tau deposition, also corroborated by the above-mentioned reports, contrasts partly with neuropathological assessments of regional tau deposition severity in *post mortem* tissue (Braak and Braak, 1991; Braak et al., 2006) where no such posterior predominance has been reported. It should be taken into account, however, that neuropathological sampling techniques commonly examine selected slices from different brain regions and are not necessarily comparable to an averaged quantification of tau PET ligand uptake within a specific brain region. Moreover, the distinct distribution profile of AV-1451 may be determined by a specific binding to certain types of tau aggregates, such as indicated by a recent study showing a relatively selective binding of AV-1451 to neurofibrillary tangles as compared to the paired helical tau filaments found in neuropil threads and neuritic plaques (Braak et al., 1986; Ono et al., 2017).

Nonetheless, tau deposition in AD might exhibit regional non-linearity, occurring early in allocortical regions of the MTL, but in greater absolute amounts at later disease stages in isocortical areas. This is in line with the here observed relatively more pronounced involvement of MTL/limbic subnetworks (e.g., 9, Temporal pole-Anterior MTL LIM and 15, Posterior MTL-retrosplenial DMN) in the more prodromal ADNI sample compared to BioFINDER sample (see Figure 4). Moreover, in our study tau deposition was not quantified absolutely but relative to cognitively healthy controls, a group that has previously been reported to exhibit significant tau deposition in MTL and related limbic circuits (primary age-related tauopathy, PART; Crary et al., 2014; Schöll et al., 2016), which may result in an underestimation of absolute MTL/limbic AV-1451 retention in patient groups as represented in our mean Z-maps.

On a methodological note, tau-sensitive PET ligands might exhibit regionally differing saturation rates of ligand uptake, leading to over- or underestimation of actual regional tau pathology by interpretation of tau PET data; however, this has hitherto only been reported for another tau PET ligand, not AV-1451 (Lemoine et al., 2015; Marquié et al., 2015; Smith et al., 2016). In addition, several nuisance factors could have influenced our results and have not been taken into account when interpreting our results. Both study samples were relatively small and were not matched for age, education level, or disease severity. We used plain Z-scores to examine spatial AV-1451 retention patterns, not adjusting for any covariates which might have affected group differences.

According to our present findings, AD-typical tau deposition predominantly targets higher-order cognitive networks over primary sensory-motor networks, but is not specific for the DMN or any other single large-scale functional brain network as a whole. This finding has implications for popular models of network specific spread of tau pathology derived from observations of prion-like mechanisms of transsynaptic tau transmission in animal models. According to these models, tau deposition within a given seed region would primarily spread within the interconnected network of this brain region, before it spreads to separate networks (Fox et al., 2011; Liu et al., 2012; Spires-Jones and Hyman, 2014; Menkes-Caspi et al., 2015). Our finding of a disproportionate affection of specific submodules within large-scale functional brain networks could be explained by a combination of (i) transsynaptic spread process and (ii) inherent regional differences in the vulnerability to tau accumulation. Tau may spread to all intrinsic network connections of one specific seed region, but only accumulate in those areas that exhibit an inherent susceptibility to tau aggregation, possibly determined by its specific functional role and molecular architecture (Zhou et al., 2012; Freer et al., 2016). Furthermore, our observation of a fairly parallel affection of several distinct (higher-order) brain networks in AD may point to the existence of several parallel seed regions of tau progression, rather than a single disease “epicenter” (Seeley et al., 2009; Zhou et al., 2012).

In conclusion, the present study indicates that the regional deposition of hyperphosphorylated tau aggregates in AD does not specifically target the default-mode or related limbic networks, but more generally affects higher-order cognitive over primary sensory-motor networks.

## Author contributions

MS, MG, and OH designed the study; TS and MS conducted data preprocessing; MG and MS performed data analyses; OH, TO, DH, JJ, and RS contributed with data acquisition; MS and MG drafted the manuscript; all authors revised and approved the final manuscript.

### Conflict of interest statement

Dr. Hansson has served at advisory boards for Eli Lilly and received research support from GE Healthcare and Hoffmann LaRoche. The other authors declare that the research was conducted in the absence of any commercial or financial relationships that could be construed as a potential conflict of interest.

## Supplementary material

Supplementary Figure 1 **Intrinsic functional brain network segmentation based on rs-MRI data (Yeo et al., **2011**)**.
