# Supplementary figures and images for: Tau Pathology Distribution in Alzheimer's disease Corresponds Differentially to Cognition-Relevant Functional Brain Networks
Source: Front Neurosci. 2017 Mar 31;11:167. doi: 10.3389/fnins.2017.00167 (PMC5374886; doi:10.3389/fnins.2017.00167)

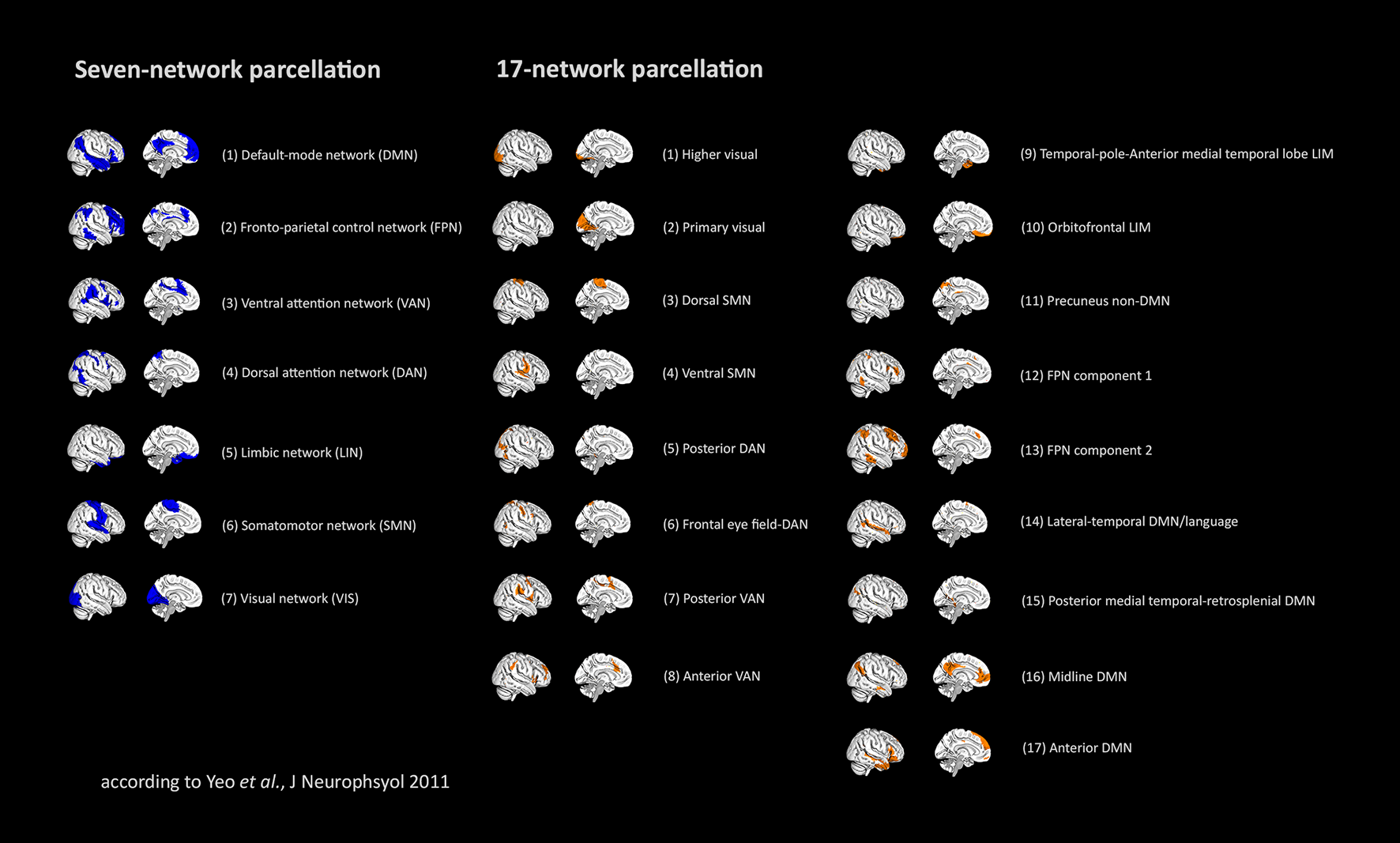

Supplement: Supplementary Figure 1 — Intrinsic functional brain network segmentation based on rs-MRI data (Yeo et al., 2011). [file Image1.TIF]
